# Lactylation at the crossroads of metabolism and epigenetics in neuroinflammation

**DOI:** 10.3389/fimmu.2026.1784112

**Published:** 2026-04-02

**Authors:** Haoyue Wang, Kangmei Shao, Ran Zhou, Jiajia Xi, Wenjing Zhang, Yazhen Hao, Jingjing Song, Zhaoming Ge

**Affiliations:** 1 Department of Neurology, Lanzhou University Second Hospital, Lanzhou, China; 2 Department of Ophthalmology, Lanzhou University Second Hospital, Lanzhou, China; 3 Institute of Pharmacology, School of Basic Medical Sciences, Lanzhou University, Lanzhou, China; 4 Department of Neurology, Qinghai Provincial People’s Hospital, Xining, China; 5 The Second Clinical Medical College, Lanzhou University, Lanzhou, China

**Keywords:** epigenetic regulation, ischemic stroke, lactylation, microglia, neuroinflammation

## Abstract

Lactate has moved from being viewed as an inert glycolytic end-product to a pleiotropic metabolite that shapes cellular signaling and gene regulation. A major inflection point is the identification of lysine lactylation (Kla), a post-translational modification that can couple glycolytic state to chromatin remodeling and protein function. In the central nervous system, lactate production, compartmentalization, and transport—coordinated by cell-type–specific expression of lactate dehydrogenases and monocarboxylate transporters within the neurovascular unit—create dynamic microenvironments that are increasingly recognized as determinants of neuroinflammatory tone. Emerging evidence indicates that Kla occurs on both histone and non-histone substrates and can reprogram inflammatory and stress-response networks in microglia, astrocytes, endothelial cells, and neurons, intersecting with canonical pathways such as NF-κB, inflammasome signaling, and cytokine-driven transcriptional programs. However, the field faces key mechanistic and translational gaps, including incomplete definition of Kla “writers/erasers/readers,” uncertainty about the quantitative relationship between lactate flux and site-specific lactylation, and marked context dependence across disease stage, cell state, and brain region. This review integrates current understanding of CNS lactate metabolism and trafficking with the expanding landscape of Kla biology, synthesizes cell- and disease-specific evidence across acute injury and neurodegeneration, and outlines priorities for causal mapping, biomarker development, and time-windowed, cell-targeted therapeutic strategies that attenuate maladaptive inflammation without compromising repair.

## 1 Introduction

Neuroinflammation is a defining pathological process across diverse neurological disorders, including ischemic stroke, traumatic brain injury, Alzheimer’s disease, Parkinson’s disease, and multiple sclerosis (1). While transient and tightly regulated inflammatory responses can support tissue repair, persistent or dysregulated inflammation disrupts neural homeostasis, compromises blood–brain barrier integrity, and accelerates neuronal loss (2, 3). Activated microglia, reactive astrocytes, endothelial cells, and infiltrating immune cells establish a complex inflammatory milieu in which metabolic state emerges as a critical determinant of cellular phenotype and outcome. Understanding how metabolic changes interface with immune regulation in the central nervous system (CNS) has therefore become a major focus of contemporary neurobiology (4, 5).

Traditionally viewed as a byproduct of glycolysis, lactate is now recognized as a multifunctional metabolite that bridges metabolism, signaling, and epigenetic control (6, 7). Under hypoxic or inflammatory conditions, glycolytic reprogramming in glia and neurons leads to dynamic lactate production, which is transported across the neurovascular unit by monocarboxylate transporters (MCT1, MCT2, and MCT4) to sustain neuronal energy supply and remodel the microenvironment (8–11). Beyond serving as an energy substrate, lactate also functions as a signaling metabolite that modulates neuronal activity and supports neurovascular coupling through both HCAR1-dependent and HCAR1-independent mechanisms. By reshaping cellular metabolic programs and lactate handling, HCAR1-mediated signaling may further alter intracellular lactate availability, thereby influencing the lactate pools that enable lysine lactylation (12–15).

A paradigm shift occurred with the discovery of lysine lactylation (Kla), a post-translational modification first identified on histones but increasingly observed in non-histone proteins (16, 17). Kla provides a direct biochemical mechanism by which lactate accumulation influences chromatin structure and transcriptional activity. Evidence now indicates that lactylation shapes inflammatory gene expression in microglia (18), astrocytes (19, 20), neurons (21), and endothelial cells (22) by intersecting with key pathways such as NF-κB (23, 24), NLRP3 (25), HIF-1α (26), and STATs (22). In particular, microglial histone lactylation has been linked to pro-inflammatory cascades (27), whereas astrocytic lactate release may supply both metabolic and epigenetic substrates that influence neuronal and vascular responses (19). Although still at an early stage, these findings establish lactylation as a metabolic–epigenetic switch in neuroimmune regulation, providing a conceptual framework for understanding how metabolic stress translates into inflammation and neurodegeneration.

## 2 Lactate and lactylation in the CNS

### 2.1 Lactate metabolism and transport in the CNS

Glucose is metabolized through glycolysis to pyruvate, which under aerobic conditions enters the tricarboxylic acid (TCA) cycle, but under hypoxic, ischemic, or inflammatory states is reduced to lactate via lactate dehydrogenase (LDH) (28–30). The brain contributes significantly to systemic lactate metabolism, generating about 13% of circulating lactate at rest and markedly increasing its uptake during exercise (31). Lactate exists in two stereoisomeric forms: L-lactate, the predominant product of glycolysis, and D-lactate, which arises mainly from the glyoxalase pathway or from gut microbiota (32). Clearance occurs either through oxidation of lactate back to pyruvate for re-entry into the TCA cycle or through conversion to glucose in the liver and muscle via the Cori cycle (33). Together, these processes establish a dynamic balance of lactate production, utilization, and recycling that underpins its role in CNS energy metabolism. The brain has minimal energy reserves, requiring tight coupling between neuronal activity and metabolism. Distinct metabolic signatures of neural cells necessitate metabolite exchange, most notably between astrocytes and neurons. Astrocytes rely on glycolysis and produce L-lactate, which is shuttled to neurons to sustain energy supply, maintain redox balance, and modulate neurotransmission. Disruption of this astrocyte–neuron metabolic cooperation contributes to neurological disease, underscoring lactate’s central role as both an energy substrate and a signaling mediator within the CNS. This astrocyte–neuron lactate shuttle is essential for excitability and plasticity (34). Moreover, under inflammatory conditions, microglia undergo metabolic reprogramming toward glycolysis, generating elevated lactate levels that contribute to the regional lactate burden (35, 36).

**Figure 1:**
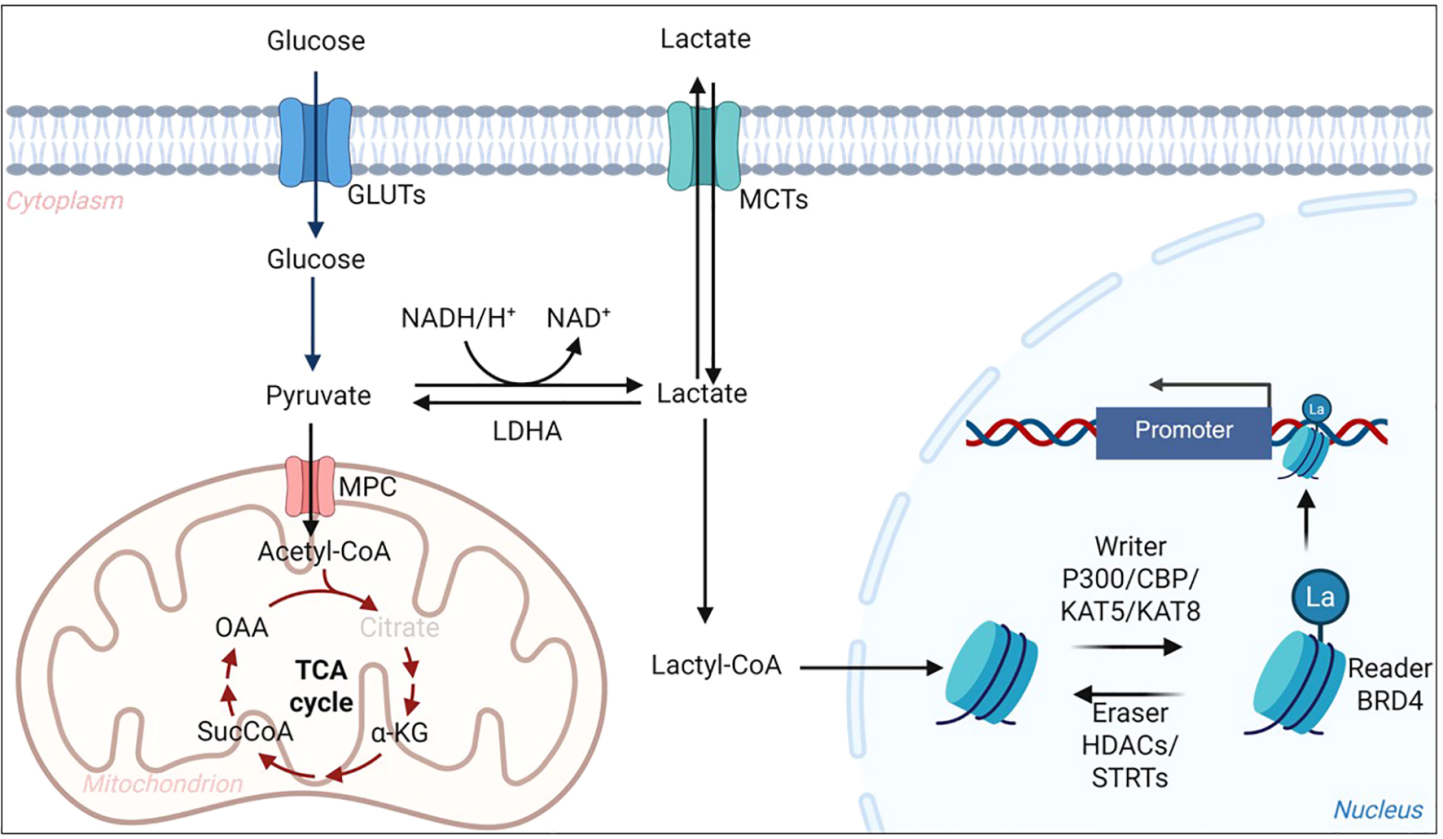
Overview of lactate metabolism and lysine lactylation in cells. Glucose enters cells via GLUTs and is converted to pyruvate through glycolysis. Pyruvate is either oxidized in mitochondria via MPC and the TCA cycle or reduced to lactate by LDHA. Lactate can be exchanged between cells through MCTs and converted into lactyl-CoA, which serves as a donor for histone and non-histone lactylation. p300/CBP and KAT5/KAT8 act as writers to transfer lactyl groups, while HDACs and SIRTs function as erasers to remove them. This pathway links glycolytic flux to epigenetic regulation and inflammatory gene expression in the CNS. GLUT, glucose transporter; MPC, mitochondrial pyruvate carrier; LDHA, lactate dehydrogenase A; MCT, monocarboxylate transporter; TCA, tricarboxylic acid; Acetyl-CoA, acetyl–coenzyme A; Lactyl-CoA, lactyl–coenzyme A; HDAC, histone deacetylase; SIRT, sirtuin; CBP, CREB-binding protein; KAT5/KAT8, lysine acetyltransferases 5/8; CNS, central nervous system.

The distribution of lactate in the CNS is governed by specialized transport systems. Monocarboxylate transporters (MCTs) mediate its bidirectional movement along concentration gradients (37). MCTs are extensively expressed throughout the brain. MCT1, enriched in astrocytes and endothelial cells, facilitates export and blood–brain barrier exchange; MCT2, predominantly neuronal, ensures rapid uptake as an oxidative substrate; and MCT4, expressed in glycolytically active astrocytes and microglia, primarily drives efflux under inflammatory or hypoxic stress. This complementary expression pattern coordinates lactate shuttling within the neurovascular unit (38). Structurally, MCTs are predicted to contain 12 transmembrane helices with intracellular C- and N-termini and operate through proton-coupled conformational changes that allow passive, bidirectional transport of monocarboxylates (38, 39). Isoform-specific kinetics confer distinct physiological roles: MCT2 exhibits the highest affinity for lactate (Km 0.5–0.75 mM) (40), MCT1 shows intermediate affinity (Km 3.5–10 mM) (41), whereas MCT4 has a low affinity but high turnover rate (Km 22–28 mM), making it particularly suited for lactate efflux during glycolytic stress (39, 41, 42). Beyond lactate, MCTs also transport pyruvate, ketone bodies, and pharmacological agents such as 3-bromopyruvate and dichloroacetate (42, 43). Importantly, MCT activity is dynamically regulated. Neuronal MCT2 expression can be rapidly upregulated at synaptic sites by neuroactive signals such as BDNF, insulin, or noradrenaline, ensuring adequate lactate supply during heightened activity (43–45). Pathological alterations in MCTs contribute to disease: reduced MCT1 expression in oligodendrocytes underlies axonal degeneration in ALS (46), loss of neuronal MCT2 is associated with cognitive decline in Alzheimer’s models (47), and abnormal redistribution or downregulation of MCTs has been described in epilepsy and ischemia (48–51). Such evidence underscores that MCTs not only mediate metabolic shuttling under physiological conditions but also represent critical determinants of vulnerability or resilience in neurological disorders (**Figure 1**).

In parallel, lactate also acts through receptor-mediated signaling. Hydroxycarboxylic acid receptor 1 (HCAR1/GPR81), a G-protein–coupled receptor expressed in neurons, astrocytes, and endothelial cells, regulates cyclic AMP signaling, vascular tone, and immune activation (52–54). Importantly, HCAR1 signaling and MCT-mediated lactate transport should not be viewed as disconnected systems. MCTs directly control intracellular lactate availability that can feed lactyl-CoA generation and lysine lactylation, whereas HCAR1-dependent signaling may indirectly modulate the propensity for intracellular lactylation by reshaping cellular metabolic state and lactate handling, including glycolytic flux and lactate transport dynamics. Thus, receptor- and transporter-mediated lactate sensing can converge on intracellular lactate pools that support Kla and downstream transcriptional and proteomic remodeling.

### 2.2 lactylation in the CNS

Kla is a recently identified post-translational modification in which a lactyl group is covalently attached to the ϵ-amino group of lysine residues (16). First characterized on histones, Kla alters nucleosome architecture and promotes transcriptional activation (16, 55). Biochemically, Kla may arise via both enzymatic and non-enzymatic routes. Lactyl-CoA, derived from intracellular lactate, is a plausible donor substrate for candidate acyltransferase-like enzymes (e.g., p300/CBP in specific contexts); however, the physiologically dominant “lactyltransferase” machinery and its substrate specificity remain incompletely defined (56, 57). In parallel, spontaneous non-enzymatic lactylation can occur when intracellular lactate or acyl-CoA pools accumulate during metabolic stress (58, 59). Importantly, emerging evidence suggests that Kla is not restricted to pathological settings but may also participate in physiological CNS adaptation, in which lactate flux and cellular redox state act as metabolic cues to tune transcriptional programs and protein function in an activity- and cell type–dependent manner (60). In this framework, lactate generated by astrocytes or neurons can be shuttled through MCTs to support energy demand while simultaneously engaging HCAR1-dependent and -independent signaling pathways that converge on activity- and stress-responsive networks, thereby providing a plausible mechanistic link between lactate dynamics and protein lactylation under homeostatic conditions (9, 13, 15).

Beyond histones, proteomic studies have revealed widespread non-histone lactylation, affecting proteins involved in metabolism (e.g., glycolytic enzymes), cytoskeletal regulation, and immune signaling (61–64). These modifications may influence enzyme activity, protein–protein interactions, or protein stability, extending the role of Kla beyond transcriptional control. Importantly, lactylation is dynamic and rises in parallel with glycolytic flux, positioning it as a rapid biochemical conduit through which metabolic perturbations are translated into altered cellular states (65). This dual capacity to regulate both chromatin and non-chromatin targets underscores its versatility in CNS physiology and pathology. Notably, disease-oriented mechanistic studies in the CNS remain skewed toward a limited set of histone marks, particularly H3K18la and H4K12la, whereas non-histone lactylation targets are emerging but are less comprehensively mapped and functionally validated.

Histone and non-histone lactylation likely represent two coordinated layers translating lactate accumulation into phenotype. Histone Kla mainly functions as a slower “transcriptional programming” mechanism that stabilizes inflammatory or repair gene expression, whereas non-histone Kla provides a faster “proteome-tuning” route by directly altering enzyme activity, protein interactions, and signaling outputs. Proteomic studies indicate that non-histone lactylation is enriched in metabolic enzymes and signaling proteins, supporting feed-forward amplification of glycolysis and rapid rewiring of inflammatory pathways under stress. Together, these layers help explain how lactate can drive both immediate functional changes and sustained transcriptional reconfiguration in CNS pathophysiology.

### 2.3 Regulation and detection

The regulation of Kla is increasingly recognized as reversible. Evidence suggests that histone deacetylases (HDACs) and sirtuins may act as “erasers,” removing lactyl groups from lysine residues (66, 67). Recent work further identified SIRT3 as a specific delactylase for H3K9la, directly removing this mark and repressing tumor-promoting gene transcription in esophageal squamous cancer cells (67). Although the substrate specificity of individual isoforms remains poorly defined, these findings indicate that lactylation, like acetylation, is subject to enzymatic control. Such reversibility enables rapid adaptation of the epigenetic landscape to metabolic changes and raises the prospect of pharmacological modulation.

Technological progress has facilitated detection of Kla, though significant challenges remain. Mass spectrometry–based proteomics is the gold standard for site-specific mapping, often combined with enrichment strategies. Pan-Kla and site-specific antibodies enable immunoblotting and immunofluorescence, including analysis of histone marks such as H3K18la and H4K12la (68). However, antibody specificity is variable, and cross-reactivity with other acyl modifications is a recurring issue. Emerging methods—single-cell proteomics, spatial epigenomics, and advanced imaging—promise greater resolution of Kla dynamics in CNS tissues.

Taken together, current advances establish lactylation as a reversible and regulatable modification. Yet methodological refinements are essential to capture its temporal, spatial, and cell type–specific patterns, which will be critical for clarifying its contribution to neuroinflammation.

## 3 Cell-specific roles in neuroinflammation

**Figure 2:**
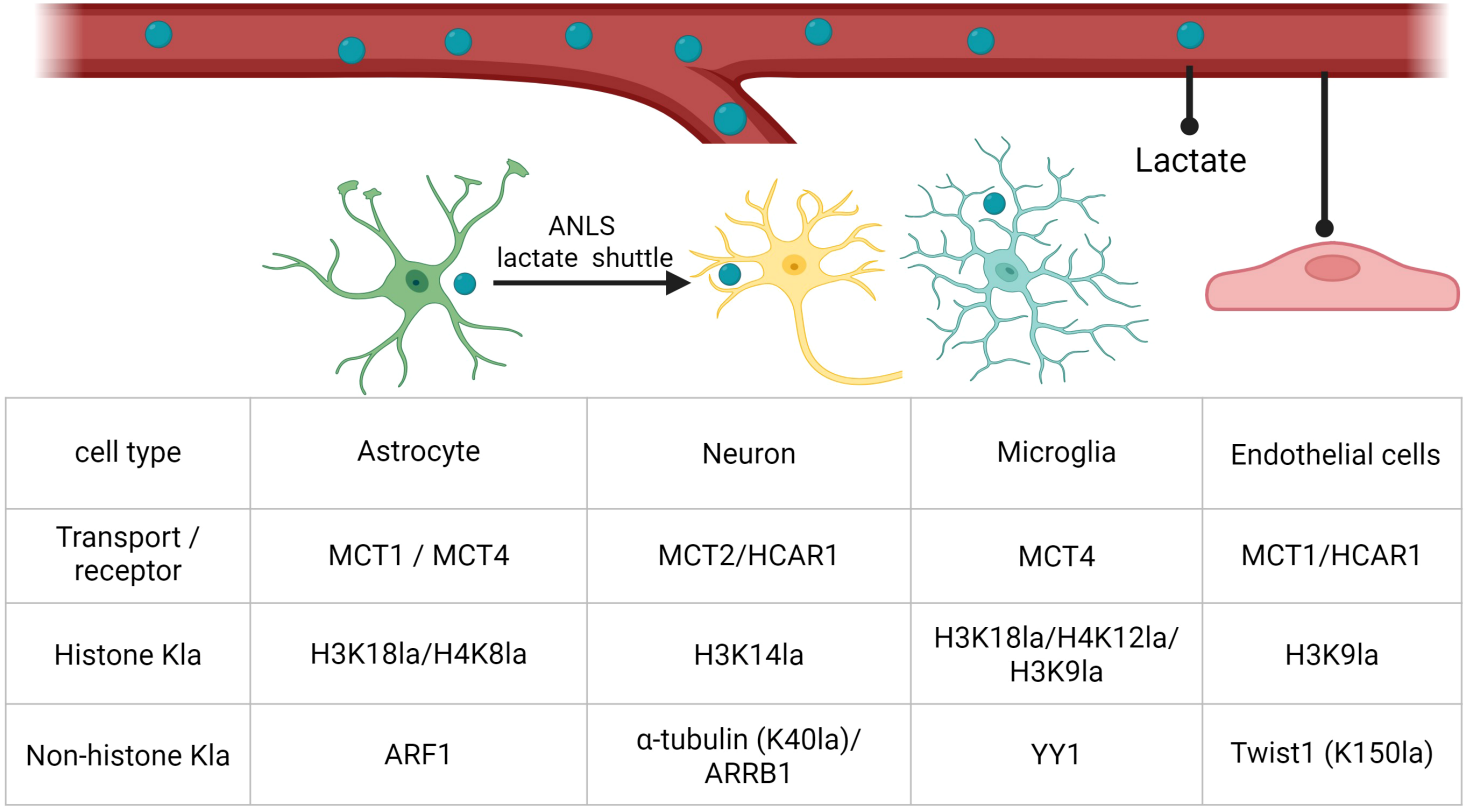
Representative transporters, receptors, and histone versus non-histone lactylation targets across CNS cell types. Representative lactate transport/receptor components and selected histone and non-histone lysine lactylation (Kla) targets in astrocytes, neurons, microglia, and endothelial cells. Abbreviations: MCT, monocarboxylate transporter; HCAR1, hydroxycarboxylic acid receptor 1; Kla, lysine lactylation; H3K18la/H4K8la/H4K12la/H3K9la/H3K14la, histone H3 or H4 lysine lactylation at the indicated residue; ARF1, ADP-ribosylation factor 1; α-tubulin (K40la), α-tubulin lysine-40 lactylation; ARRB1, β-arrestin 1; YY1, Yin Yang 1; Twist1 (K150la), Twist1 lysine-150 lactylation.

In the neurovascular unit, lactate is not a uniform signal but a compartmentalized metabolite whose handling differs across cell types. These differences shape intracellular lactate availability and, consequently, the balance between histone and non-histone lysine lactylation programs that tune inflammatory transcription, cell-state transitions, and intercellular crosstalk (**Figure 2**).

### 3.1 Microglia

Microglia, the resident immune cells of the CNS, are central mediators of neuroinflammation (69). Upon activation by ischemia, trauma, infection, or protein aggregates, microglia undergo profound metabolic reprogramming characterized by a shift toward aerobic glycolysis (70–72). This metabolic state not only sustains rapid energy demands but also leads to lactate accumulation, fueling Kla. Histone Kla—particularly H3K18la and H4K12la—has been linked to transcriptional activation of NF-κB–dependent mediators, NLRP3 inflammasome components, and hypoxia-inducible factor 1α (HIF-1α) targets, suggesting a role in amplifying pro-inflammatory cascades (18, 24, 73). At the same time, lactylation may influence microglial polarization (74). Although elevated Kla often associates with pro-inflammatory phenotypes, in other immune cells such as macrophages, some evidence indicates that it also contributes to resolution by supporting transcription of reparative or anti-inflammatory genes (6). This dual role underscores the context dependence of Kla, in which timing and intensity of metabolic stress shape microglial outcomes. Together, these findings highlight microglial lactylation as a pivotal metabolic–epigenetic mechanism that translates glycolytic reprogramming into neuroimmune phenotypes, making it a promising therapeutic target for modulating inflammation.

In AD, microglia show enhanced glycolysis and elevated histone lactylation, notably H4K12la and H3K18la, which reinforce maladaptive activation states. Mechanistically, these modifications establish positive feedback loops: H4K12la amplifies glycolytic gene transcription through PKM2, driving persistent pro-inflammatory activation and cognitive decline, while H3K18la enhances NF-κB signaling to promote SASP factors such as IL-6 and IL-8, linking microglial senescence to neurodegeneration (18, 24). Environmental stressors such as cigarette smoke further exacerbate this process by increasing lactate production and H4K12la at the NLRP3 promoter, promoting autophagy dysfunction and plaque accumulation (25). These studies highlight how metabolic–epigenetic coupling in microglia drives AD progression.

Beyond AD, similar lactylation-mediated mechanisms operate across a spectrum of neurological diseases. In hypoxic-ischemic encephalopathy (HIE), increased H3K9la promotes M1 polarization via TNF signaling (75), whereas in Parkinson’s disease (PD), H3K9la at the SLC7A11 promoter drives glutathione metabolism and neuroinflammation (73). Stroke models reveal context-dependent outcomes: in acute ischemia, lactate overload elevates H3K9la and aggravates neuroinflammation via SMEK1 deficiency (26), yet in other studies, H3K18la promoted anti-inflammatory reprogramming and neuroprotection through plxnb2 transcription (76). This apparent duality suggests that Kla’s impact may be dictated by timing, local metabolic environment, and target gene networks. Such paradoxical roles underscore the need for spatiotemporal resolution of Kla dynamics in microglia.

Microglial lactylation is not confined to histones. Non-histone targets such as the transcription factor YY1 have been identified in ocular angiogenesis and autoimmune uveitis, where its lactylation enhances FGF2 or inflammatory gene expression, fueling pathological outcomes (27, 77). Similarly, lactate-induced H4K12la promotes PD-1 transcription in microglia after spinal cord injury (SCI), facilitating repair processes such as axon regeneration and functional recovery (78, 79). Other studies converge on glycolytic remodeling and lactylation as critical modulators of the immune milieu, influencing CXCL16-mediated T cell recruitment (80). Collectively, these findings reveal that microglial lactylation serves as a molecular rheostat, capable of either propagating inflammation or promoting repair, depending on pathological stage and signaling axis involved.

Exercise and systemic metabolic factors also shape microglial lactylation. Running-induced lactate, or exogenous sodium lactate, can bias microglia toward a reparative phenotype and improve cognition, suggesting physiological lactate may act as a beneficial “accelerator” of phenotype transition (81). Conversely, anesthetic exposure (sevoflurane) reduces histone lactylation and disrupts microglial regulatory pathways, aggravating pyroptosis and cognitive deficits in neonatal models (82). These observations illustrate that lactylation is highly sensitive to systemic metabolic cues and environmental exposures, reinforcing the concept that microglia integrate local and systemic metabolic states through Kla.

Taken together, microglial lactylation emerges as a unifying mechanism that links metabolic reprogramming to transcriptional control across neurodegenerative, ischemic, autoimmune, developmental, and toxicological contexts. Yet the consequences of this modification are highly context-dependent, capable of driving either deleterious inflammation or beneficial repair. A deeper mechanistic understanding—particularly of non-histone lactylation, isoform-specific “writers” and “erasers,” and spatiotemporal regulation—is needed to translate these insights into therapeutic interventions.

### 3.2 Astrocytes

Astrocytes, as the most abundant glial population in the CNS, exhibit remarkable metabolic plasticity that positions them as central hubs in lactate production and lactylation-dependent regulation of neuroinflammation (83). Under physiological conditions, astrocytic glycolysis supports the astrocyte–neuron lactate shuttle, thereby sustaining neuronal activity (9, 84, 85). However, under pathological stress such as ischemia, trauma, or neurodegeneration, astrocytes undergo profound glycolytic reprogramming, markedly elevating lactate output and driving histone and non-histone lactylation cascades (19, 86–88).

A growing body of evidence indicates that astrocytic histone lactylation serves as a transcriptional switch coupling metabolic flux to immune activation. In cerebral ischemia–reperfusion injury, lactate accumulation promotes H3K18la-mediated activation of NSUN2, enhancing m^5C RNA modification and reinforcing astrocytic proinflammatory programs (86). Similarly, in bilirubin encephalopathy, H3K18la at the NOD2 promoter activates downstream MAPK and NF-κB signaling, exacerbating astrocytic pyroptosis and amplifying inflammatory cascades (87). In spinal cord injury, a UCHL1/PFKFB3/H4K8la positive feedback loop drives persistent glycolysis, sustained lactate production, and self-reinforcing lactylation, which fuels both astrocytic reactivity and secondary tissue damage (88). These mechanistic insights converge on a theme: astrocytic lactylation not only reflects metabolic stress but also actively shapes transcriptional networks that worsen neuroinflammatory pathology. Yet astrocytic lactylation is not uniformly detrimental. Following subarachnoid hemorrhage, BRD4-dependent regulation of H4K8la was shown to restrain maladaptive A1 astrocytic polarization, suggesting that appropriate levels of astrocytic lactylation may bias glial states toward functional recovery (89). Conversely, excessive lactate release during ischemia aggravates neuronal death and glial activation, with astrocyte-specific LDHA knockout or pharmacological inhibition of lactylation markedly alleviating injury (20). These contrasting findings underscore the context- and time-dependent roles of astrocytic lactylation, which may be deleterious in acute ischemia yet beneficial during recovery or resolution phases.

Importantly, astrocytic lactylation extends beyond histones to non-histone proteins, influencing cross-cellular communication. In ischemic stroke, astrocytic LRP1 suppresses glycolysis and reduces ARF1 lactylation, thereby enabling astrocyte-to-neuron mitochondrial transfer and protecting neurons (19). Such findings highlight that lactylation is not merely an epigenetic mark but also a molecular determinant of astrocytic crosstalk with neurons and endothelial cells, shaping outcomes at the neurovascular unit.

Collectively, these studies establish astrocytic lactylation as a metabolic–epigenetic nexus with dualistic roles. On the one hand, astrocytic glycolysis and histone lactylation amplify inflammatory cascades through NF-κB, NOD2, and RNA methylation pathways (87, 88). On the other hand, regulatory lactylation circuits can promote neurovascular repair or maintain neuronal energy balance (19, 89). This paradox suggests that astrocytic lactylation may function as a rheostat, with its outcome determined by the intensity, duration, and cellular context of metabolic stress.

### 3.3 Endothelial cells

Endothelial cells, forming the blood–brain barrier (BBB), contribute to neuroinflammation by regulating vascular permeability, leukocyte adhesion, and cytokine signaling (90). Recent studies outside the CNS have provided direct evidence that lactylation in ECs shapes angiogenesis, inflammation, and fibrosis (91–94). For example, VEGF stimulation increases glycolysis and lactate production in ECs, leading to enrichment of H3K9la at promoters of angiogenic genes. This lactylation event establishes a positive feedback loop with HDAC2, thereby sustaining transcriptional programs required for neovascularization (91). In diabetic retinopathy, chronic hyperglycemia elevates lactate-mediated histone lactylation, which upregulates the m^6A demethylase FTO, enhancing angiogenic and inflammatory phenotypes in retinal ECs and promoting vascular leakage and neurodegeneration (92). These findings indicate that lactylation can bridge metabolic reprogramming with epigenetic and post-transcriptional regulation to reinforce pathological microvascular remodeling. Beyond angiogenesis, lactylation in ECs contributes to inflammatory and fibrotic processes. In sepsis, lactate accumulation promotes lactylation of macrophage-derived CIRP, which is subsequently internalized by pulmonary vascular ECs to stabilize ZBP1, activating RIPK3-dependent PANoptosis and thereby exacerbating acute lung injury (93). Similarly, under ischemic conditions in skin flap models, PKM2-driven glycolytic lactate production induces Twist1 lactylation (K150la) in ECs, promoting its phosphorylation, nuclear translocation, and transcriptional activation of TGF-β1. This lactylation-dependent pathway facilitates EndoMT and fibrosis, linking endothelial metabolism to tissue scarring (94). Together, these diverse contexts highlight that EC lactylation is not limited to angiogenesis but also mediates cell death, fibrosis, and vascular dysfunction across systemic diseases.

While the evidence from peripheral vascular beds is compelling, studies specifically addressing lactylation in brain endothelial cells remain scarce. Nevertheless, these systemic findings strongly suggest that CNS endothelium at the blood–brain barrier (BBB)—which is similarly glycolysis-dependent and exposed to astrocytic and microglial lactate flux—may undergo analogous lactylation-driven reprogramming. Such mechanisms could influence tight junction regulation, leukocyte adhesion, and BBB breakdown in neurological disorders. Future work employing single-cell multi-omics and *in vivo* lactylation mapping at the neurovascular interface will be essential to clarify whether the angiogenesis- and fibrosis-related lactylation circuits described in peripheral endothelium are recapitulated in CNS pathology.

### 3.4 Neurons

Neurons, although predominantly oxidative, integrate lactate not only as an alternative energy substrate but also as a key signal for post-translational and epigenetic regulation. Accumulating evidence shows that lactate-derived Kla in neurons reshapes cytoskeletal dynamics, transcriptional programs, and survival pathways, thereby influencing neuronal plasticity and vulnerability in both physiological and pathological settings. One of the most striking findings is that neuronal lactylation extends beyond histones to cytoskeletal proteins. HDAC6-catalyzed α-tubulin lactylation at lysine 40 was shown to enhance microtubule dynamics and neurite outgrowth, linking glycolytic flux to structural remodeling in hippocampal neurons (95). Beyond cytoskeletal regulation, neuronal histone lactylation modulates transcriptional circuits in disease-relevant contexts. For example, in the hypothalamus, increased H4K12la in pro-opiomelanocortin (POMC) neurons promoted neuroprotective and anti-obesity transcriptional programs, highlighting lactylation as a metabolic–epigenetic regulator of neuronal fate and systemic energy balance (21). Similarly, under hypoxia, lactate-induced H3K9la in neural stem/progenitor cells activated SnoN-dependent transcription and promoted neuronal differentiation, suggesting a developmental role for Kla in neurogenesis (96). In contrast, pathological elevations of neuronal lactate can exacerbate injury by engaging maladaptive lactylation cascades. In intracerebral hemorrhage, lactate accumulation induced H3K14la, which transcriptionally upregulated PMCA2, elevated intracellular calcium, and promoted ferroptotic death of cortical neurons (97). In subarachnoid hemorrhage, p300-driven lactylation of β-arrestin1 (ARRB1) enhanced S100A9 expression, triggering mitochondrial dysfunction and apoptosis (98). Similarly, in traumatic brain injury, histone lactylation increased expression of PSMD14, a proteasome regulator, which augmented mitophagy and protected neurons against PANoptosis, highlighting that lactylation can also activate compensatory neuroprotective responses depending on context (99). Neuronal lactylation has also been implicated in maladaptive plasticity. In peripheral sensory neurons, amphiregulin/EGFR signaling enhanced glycolysis and drove p300-dependent H3K18la and H4K12la, thereby upregulating pronociceptive genes and sustaining neuropathic pain (100). These findings underscore that Kla functions as a transcriptional amplifier, where metabolic shifts can durably reprogram neuronal excitability and network activity.

Taken together, neuronal lactylation emerges as a double-edged sword. On the one hand, it supports structural plasticity, neurogenesis, and metabolic homeostasis (21, 95, 96); on the other, it amplifies excitotoxic, ferroptotic, or pronociceptive programs under pathological stress (97, 98, 100). This bidirectional nature suggests that neuronal lactylation acts as a rheostat integrating metabolic state with epigenetic programming. However, most studies rely on rodent models and acute injury paradigms, with limited resolution of spatiotemporal dynamics or human validation.

## 4 Kla in neurological disorders

### 4.1 Ischemic stroke

Ischemic stroke triggers a profound metabolic crisis characterized by oxygen and glucose deprivation, forcing neurons and glia into glycolysis and producing a rapid accumulation of lactate in the infarct and peri-infarct regions (101). This metabolic shift has long been recognized as detrimental, but the discovery of lactylation—an epigenetic and post-translational modification derived from lactate—has reframed lactate as a signaling metabolite. Proteomic studies confirm that proteins undergo extensive lactylation after stroke (22). Specific events, such as non-histone lactylation of LCP1 or histone H3K18la-driven HMGB1 expression, have been shown to aggravate ischemic injury, while inhibition of these modifications alleviates neuronal apoptosis and pyroptosis (102, 103). These findings suggest that lactylation is not just a by-product of metabolic stress, but an active mediator of ischemic pathology.

One of the central mechanisms linking lactylation to ischemic stroke is its regulation of neuroinflammation. Microglia, the resident macrophages of the brain, respond rapidly to ischemia and shape the inflammatory environment (103). Elevated lactate levels increase histone H3K18la in microglia, activating NF-κB signaling and upregulating IL-6 and IL-8, thus promoting a pro-inflammatory phenotype (24). Clinical and experimental data in other ischemic contexts show that H3K18la enhances reparative gene expression (24, 104), raising the possibility that timing and context dictate whether macrophage lactylation promotes repair or exacerbates damage in stroke. Beyond microglia and macrophages, lactylation is implicated in neutrophil recruitment and adaptive immunity. Histone lactylation enhances the transcription of CXCL1 and CXCL5, potent chemokines that drive neutrophil infiltration (104, 105). These same chemokines have been linked to infarct expansion in stroke models (106, 107). Thus, lactylation may indirectly amplify secondary brain injury by orchestrating neutrophil chemotaxis and activation. In T cells, lactylation regulates lineage-determining factors, such as IKZF1 in Th17 differentiation (108) and moesin in Treg stabilization (109). Given the established contributions of Th17, Treg, and NKT subsets to post-stroke inflammation and outcome (110, 111), it is plausible that lactylation shapes adaptive immune responses in the ischemic brain, though direct evidence remains to be generated.

**Figure 3:**
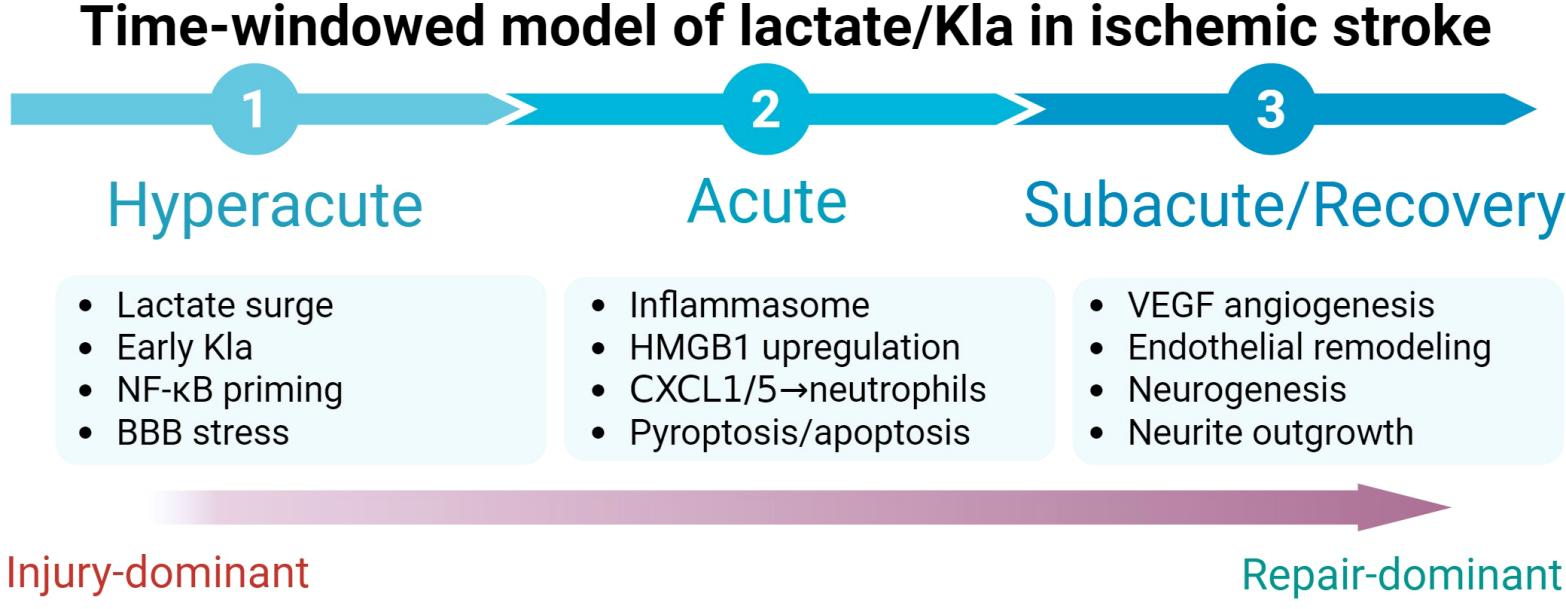
Time-windowed lactate/Kla model in ischemic stroke. Temporal schematic summarizing stage-dependent effects of lactate-driven lysine lactylation (Kla) after ischemic stroke. Hyperacute and acute phases are characterized by lactate surge and early Kla with inflammatory priming (NF-κB), inflammasome activation, chemokine-driven neutrophil recruitment (CXCL1/5), and pyroptosis/apoptosis. During the subacute/recovery phase, lactylation-associated programs are linked to vascular remodeling (VEGF/angiogenesis, endothelial remodeling) and neurorepair processes, including neurogenesis and neurite outgrowth. Abbreviations: Kla, lysine lactylation; NF-κB, nuclear factor kappa B; BBB, blood–brain barrier; HMGB1, high-mobility group box 1; CXCL1/5, C-X-C motif chemokine ligand 1/5; VEGF, vascular endothelial growth factor.

While lactylation contributes to acute injury, it may also support reparative processes during recovery. Lactylation of angiogenic regulators such as HIF-1α stabilizes the protein and enhances VEGF-mediated vascular remodeling (112, 113), and lactylation of transcription factors like YY1 can promote endothelial responses (77). Similarly, lactate-induced histone H3K9la promotes neurogenesis via SnoN during hypoxia (114), and lactate itself enhances neuronal outgrowth in OGD models (115). These findings suggest a temporal switch: in the acute phase, lactylation amplifies inflammation and barrier damage, whereas in the subacute phase it may facilitate angiogenesis and neurogenesis (**Figure 3**).

Taken together, lactylation emerges as a crucial mediator connecting metabolic stress to neuroinflammation in ischemic stroke. It influences microglial and macrophage polarization, neutrophil recruitment, T-cell differentiation, and BBB integrity. Therapeutically, targeting lactylation offers multiple intervention points: inhibiting acute injury pathways such as HMGB1 lactylation or CXCL-driven neutrophil infiltration, while preserving or enhancing later pro-repair effects on angiogenesis and neurogenesis. Although direct studies in traumatic brain injury remain limited, the shared metabolic surge, barrier disruption, and inflammatory cascades suggest that similar mechanisms may apply. Future research should focus on spatiotemporal mapping of lactylation after brain injury, mechanistic dissection in defined immune and neural subsets, and translational strategies to exploit lactylation as both a biomarker and a therapeutic target.

### 4.2 Alzheimer’s disease and neurodegeneration

Alzheimer’s disease (AD) is the most common neurodegenerative disorder, clinically manifested by progressive memory loss, cognitive decline, and behavioral disturbances (116, 117). Beyond its classical hallmarks, AD is increasingly recognized as a chronic neuroinflammatory condition (116). Aberrant activation of microglia, the resident immune cells of the CNS, plays a central role in this process, gradually shifting them from protective to dysfunctional states that exacerbate neuronal vulnerability (118). Under physiological conditions, microglia maintain CNS homeostasis, but in the diseased brain they become maladaptively activated and contribute to a self-perpetuating inflammatory milieu (119).

Recent evidence highlights histone lactylation as a critical epigenetic driver linking metabolic stress to microglial dysfunction in AD. Elevated lactate and pan-Kla, with specific enrichment of H3K18la, have been observed in senescent microglia and hippocampal tissues from aged and AD-model mice (FAD4T and APP/PS1), where H3K18la enhances NF-κB binding to the promoters of Rela (p65) and Nfκb1 (p50), thereby upregulating SASP mediators such as IL-6 and IL-8 and promoting chronic neuroinflammation (24). Complementing this axis, studies in AD patients and mice have identified increased H4K12la in Aβ plaque–associated microglia, where it is enriched at glycolytic gene promoters and establishes a glycolysis/H4K12la/PKM2 positive feedback loop that amplifies glycolytic flux and microglial activation (18). Disruption of this loop through PKM2 inhibition or microglia-specific Pkm2 deletion not only attenuates neuroinflammation but also reduces Aβ burden and improves cognition in AD models (18). Together, these findings define H3K18la/NF-κB–SASP and H4K12la/PKM2 circuits as complementary lactylation-dependent mechanisms that couple altered metabolism to persistent neuroinflammation and neurodegeneration, offering potential therapeutic entry points for AD.

### 4.3 Other neurological disorders

Beyond ischemic stroke and AD, lactate-linked lysine lactylation (Kla) has also been implicated in several other neurological disorders, mainly through cell-type–specific inflammatory reprogramming. In neonatal hypoxic–ischemic encephalopathy, oxygen–glucose deprivation increases microglial H3K9 lactylation and promotes pro-inflammatory polarization via TNF-associated signaling, linking metabolic stress to neuroinflammation (75). In Parkinson’s disease models, enhanced glycolysis-derived lactate elevates microglial H3K9la and enriches this mark at the SLC7A11 promoter in a p300/CBP-dependent manner; disrupting this axis mitigates microglia-driven inflammation and improves motor outcomes (73). In spinal cord injury, lactate-mediated lactylation has been reported to influence microglial repair programs, including PD-1–linked transcriptional regulation, supporting a stage-dependent role of Kla in balancing injury versus recovery (78). In subarachnoid hemorrhage, astrocytic histone lactylation—particularly H4K8la—can be regulated by BRD4 and modulates astrocyte polarization and neurological outcome, highlighting a distinct lactylation control point in hemorrhagic injury (89). Finally, direct evidence for non-histone lactylation in neuroimmune pathology comes from experimental autoimmune uveitis, where YY1 lactylation in microglia amplifies inflammatory gene programs and disease severity (27). Overall, while disease-specific mechanisms are emerging, systematic cell-type- and time-resolved profiling remains necessary, especially for entities where direct Kla evidence is still sparse (e.g., autoimmune encephalitides).

## 5 Mechanistic and therapeutic insights

### 5.1 Pathway intersections

Several canonical inflammatory pathways intersect with Kla, creating mechanistic links between metabolic stress and neuroimmune activation. These intersections are frequently coupled rather than parallel, such that lactylation-associated changes in one pathway can prime or reinforce others, collectively shaping the neuroinflammatory cascade. In this coupled architecture, lactylation-facilitated NF-κB priming can lower the threshold for inflammasome activation and downstream pyroptotic cell death, establishing a feed-forward inflammatory loop.

Transcriptional priming and inflammatory amplification through NF-κB. Across diverse disease settings, lactate-associated histone lactylation recurrently converges on NF-κB–dependent transcription, providing a general mechanism for sustained inflammatory output. In peripheral organs, H3K18la enrichment at the RhoA promoter activated RhoA/ROCK/Ezrin signaling and downstream NF-κB pathways in sepsis-associated acute kidney injury, aggravating inflammation and dysfunction (120). In chronic kidney disease models, glycolysis-driven lactate accumulation enhanced H4K12la at the promoters of Ikbkb, Rela, and Relb, sustaining NF-κB activation and promoting inflammation and fibrosis (23). Tumor studies similarly support histone lactylation as an amplifier of NF-κB activity, exemplified by AP001885.4–associated NF-κB activation and c-myc upregulation in esophageal squamous cell carcinoma (121). Emerging CNS evidence aligns with these systemic observations. In bilirubin encephalopathy, astrocytic H3K18la was enriched at the NOD2 promoter, activating MAPK and NF-κB signaling while exacerbating pyroptosis and neuroinflammation (87). In chronic fatigue syndrome models, neuronal PKM2-driven glycolytic reprogramming induced H4K12la, enhanced NF-κB–dependent cytokine expression, and contributed to mitochondrial dysfunction and cognitive decline (122). Together, these findings support a conserved link between lactylation and NF-κB–centered priming, while also underscoring that downstream consequences remain strongly dependent on cell state and disease stage.

Regulated cell death and autophagy as inflammatory multipliers. A key implication of NF-κB–centered priming is that it often interfaces directly with execution programs of regulated cell death and survival, making “pathway intersections” most interpretable when framed around outcomes. Evidence indicates that Kla can modulate apoptosis, pyroptosis, ferroptosis, and autophagy in a context-dependent manner (123, 124). For apoptosis, ischemic stress–associated H3K18la promoted YTHDF2 expression and cardiomyocyte apoptosis (125), whereas in endothelial cells, suppression of H3K18la reduced Apaf-1 transcription and blocked apoptosis (126). In malignancy, Kla can instead support survival, as H3K18la promoted USP39 expression and activated PI3K/AKT/HIF-1α signaling to suppress apoptosis in endometrial carcinoma (127), consistent with broader evidence that lactylation can favor immune evasion and persistence in tumor contexts (128, 129). Pyroptosis provides a direct example of opposing directionality across inflammatory milieus. In ulcerative colitis, H3K18la promoted M2 polarization and suppressed NLRP3 inflammasome activation, reducing pyroptotic injury and inflammation (130). By contrast, in ischemia–reperfusion injury, H3K18la increased HMGB1 expression and enhanced caspase-1 and GSDMD cleavage, aggravating neuronal pyroptosis and neuroinflammation (102). Ferroptosis is also intertwined with lactate/Kla circuits. In prostate cancer, inhibition of H3K18la reduced HIF-1α expression and sensitized cells to ferroptosis (113), whereas in acute lung injury, p300-mediated H3K18la upregulated METTL3 and stabilized ACSL4 to drive ferroptosis and amplify inflammation (131). Autophagy further couples metabolic–epigenetic remodeling to inflammatory persistence. H3K18la facilitated transcription of RUBCNL in chemoresistant colorectal cancer, promoting autophagosome maturation (132). In the brain, H4K12la-induced NLRP3 transcription has been linked to autophagic dysfunction in microglia, further aggravating inflammatory damage (25). Non-histone lactylation adds another layer of integration by directly modulating transcription factors. Kla-enhanced Sox10 activity promoted expression of pyroptosis-related genes including IL-1β, IL-18, and GSDMD (133, 134), while lactylated Snail1 facilitated TGF-β transcription and apoptotic signaling (134). These observations support a unified view in which lactylation coordinates transcriptional priming with cell fate programs that feed back into inflammatory injury.

Cytokine amplification through JAK/STAT signaling. Lactylation also intersects with cytokine-driven signal transduction, particularly the JAK/STAT axis. In tumors, lactate accumulation induced H3K18la and transcriptionally upregulated METTL3, enhancing m6A methylation of Jak1 mRNA and promoting JAK1–STAT3 activation that contributed to immunosuppressive myeloid phenotypes (135). In gestational diabetes mellitus, promoter hyperlactylation was enriched in JAK/STAT-related genes, suggesting a conserved regulatory link between lactylation and cytokine signaling states (136). Although CNS evidence remains limited, emerging data indicate that reduced glycolytic lactate production in microglia altered histone lactylation at the Lrrc15 promoter and affected LRRC15–CD248–JAK/STAT signaling and astrocyte differentiation (137). Given the central role of JAK/STAT, especially JAK2/STAT3, in glial activation and neuroinflammatory cascades, these findings nominate lactylation-sensitive JAK/STAT checkpoints for CNS-focused validation.

Overall, positioning NF-κB priming, regulated cell death and autophagy, and JAK/STAT cytokine signaling as an interconnected network provides a clearer account of how lactylation can produce divergent outcomes across conditions and stages. This integrated perspective also motivates therapeutic strategies centered on time-windowed and cell-targeted modulation of lactate metabolism and lactylation machinery, rather than isolated single-pathway inhibition.

### 5.2 Therapeutic strategies

Recognition of lactylation as a metabolic–epigenetic switch has opened new therapeutic opportunities. Interventions can target lactate metabolism, lactate transport/signaling, or the enzymatic machinery regulating Kla.

Critical considerations for therapeutic targeting. Lactate is not merely a pathological byproduct but a major oxidative fuel for neurons under many physiological conditions, supported by the astrocyte–neuron lactate shuttle. Therefore, indiscriminate suppression of lactate production or transport may impair baseline neuronal bioenergetics, synaptic function, and activity-dependent plasticity, and may also compromise beneficial reparative processes such as angiogenesis during recovery. A key translational challenge is to modulate pathological lactylation programs without disrupting metabolic homeostasis, favoring time-windowed and cell-targeted strategies and avoiding chronic global blockade of neuronal lactate utilization.

Metabolic modulation. Inhibitors of glycolysis such as 2-deoxy-D-glucose (2-DG) or modulators of pyruvate dehydrogenase (e.g., dichloroacetate) have been used experimentally to limit lactate accumulation and thereby reduce Kla. More specific approaches include targeting lactate dehydrogenase isoforms (LDHA/LDHB) or manipulating monocarboxylate transporters (MCTs) to fine-tune intercellular lactate flux, ideally with cell-type and stage specificity.

Epigenetic modulation. Histone deacetylase (HDAC) inhibitors, already applied clinically in cancer and epilepsy, may indirectly affect lactylation by altering acetylation–lactylation balance. Conversely, agents that selectively modulate de-lactylase activity within HDAC or sirtuin families could provide more precise control over Kla.

Receptor-mediated pathways. Hydroxycarboxylic acid receptor 1 (HCAR1/GPR81) agonists or antagonists offer a means to modulate lactate-dependent signaling with potential effects on neurovascular coupling and immune activation, and may also indirectly influence intracellular lactylation by reshaping cellular metabolic state and lactate handling. For CNS-specific delivery, advanced platforms such as nanoparticles and injectable hydrogels may facilitate targeting of lactate metabolism and lactylation regulators across the blood–brain barrier, improving therapeutic precision.

Yet, caution is warranted. Lactate can support both inflammatory injury and reparative programs; therapeutic strategies should therefore be tailored to disease stage, cell type, and metabolic context, with the goal of limiting excessive, injury-associated lactate/Kla signaling while preserving physiological lactate-dependent neuronal function.

### 5.3 Challenges

Despite rapid advances, several challenges hinder the integration of lactylation into neuroinflammation research and therapeutic development. The enzymatic machinery remains incompletely defined; although histone acetyltransferases, HDACs, and sirtuins have been implicated as potential “writers” and “erasers,” While multiple candidate enzymes have been reported to write or erase Kla in experimental settings, it remains unclear which enzymes dominate *in vivo* across cell types and disease stages, and how substrate/lysine-site specificity is achieved. This uncertainty complicates efforts to design interventions that selectively modulate lactylation without affecting other acyl modifications. Equally limiting are methodological constraints: antibody-based assays often lack specificity, while mass spectrometry, though definitive, is low-throughput and difficult to apply to clinical samples. The absence of reliable *in vivo* biomarkers further restricts the ability to track lactylation dynamics across disease stages or therapeutic interventions.

Adding to these technical hurdles is the inherent context dependence of Kla. Evidence indicates that it can amplify inflammation in acute injury while supporting reparative transcription during recovery, and in chronic neurodegeneration it may reinforce maladaptive glial activation. Such variability underscores the need for stage- and cell type–resolved analyses before clinical translation can be realized. Finally, most current insights derive from rodent or *in vitro* models, leaving its relevance to human pathology incompletely defined. Addressing these gaps will require integrated strategies that combine multi-omics profiling, spatial technologies, and functional validation to establish causal roles and unlock therapeutic potential.

## 6 Conclusion and perspectives

Lysine lactylation has reshaped our understanding of how metabolism and epigenetics converge in the inflamed brain. By linking glycolytic flux and lactate accumulation to chromatin remodeling and transcriptional control, Kla provides a mechanistic framework for how metabolic states dictate neuroimmune responses. Evidence from ischemic stroke, traumatic injury, neurodegeneration, and autoimmune encephalitides shows that lactylation in microglia, astrocytes, endothelial cells, and neurons intersects with pathways such as NF-κB, NLRP3, underscoring its role as a metabolic–epigenetic switch capable of driving both amplification and resolution of inflammation.

Despite these advances, fundamental questions remain. The enzymes responsible for writing and erasing lactyl marks, the presence of potential reader proteins, and the spatiotemporal dynamics of Kla across disease stages are still poorly defined. Bridging these gaps will require single-cell and spatial multi-omics, coupled with functional models, to establish causality and clarify context-specific roles. From a translational perspective, therapeutic strategies targeting lactylation—whether through metabolic modulation, transporter regulation, or manipulation of epigenetic enzymes—hold promise but must be applied with precision, given the dual nature of lactate in both injury and repair.

Ultimately, lactylation represents more than a biochemical curiosity; it embodies a new conceptual axis in neuroimmunology. By integrating metabolic flux with epigenetic programming, it offers not only mechanistic insight into neuroinflammation but also a potential entry point for precision therapies. Elucidating how Kla operates across cell types, disease contexts, and temporal scales may open the door to interventions that selectively reprogram maladaptive inflammation while preserving or even enhancing reparative responses in the injured and aging brain.
